# Merkel Cell Carcinoma of the Skin: Deducing the Pattern of Spread from an International Aggregated Database of 949 Patients

**DOI:** 10.3390/curroncol32040211

**Published:** 2025-04-02

**Authors:** Patricia Tai, Kurian Joseph, Vimal H. Prajapati, Aoife Jones Thachuthara, Jidong Lian, Avi Assouline, Edward Yu, Michael Veness

**Affiliations:** 1 Department of Oncology, University of Saskatchewan, Saskatoon, SK S7N 5A2, Canada; 2 Department of Oncology, University of Alberta, Edmonton, AB T6G 2R3, Canada; kurian.joseph@albertahealthservices.ca; 3 Department of Dermatology, University of Calgary, Calgary, AB T2N 1N4, Canada; vimal.h.prajapati@dermatologyphilosophy.com; 4 Department of Medical Oncology, Cork University Hospital, T12 DC4A Cork, Ireland; aoife.jones3@hse.ie; 5 Department of Oncology, University of Toronto, Toronto, ON M5S 1A1, Canada; jidong.lian@thp.ca; 6 Department of Radiation Oncology, Centre de Cancérologie de la Porte de Saint-Cloud (CCPSC), 92100 Boulogne-Billancourt, France; avi.assouline@ccpsc.fr; 7 Department of Oncology, Western University, London, ON N6A 3K7, Canada; eyu@uwo.ca; 8 Department of Oncology, University of Sydney, Sydney, NSW 2050, Australia; michael.veness@health.nsw.gov.au

**Keywords:** Merkel cell carcinoma, distant metastases, lymph node metastases, pattern of spread, skin, database

## Abstract

(1) Background: It is controversial if Merkel cell carcinomas (MCCs) spread to lymph nodes or distant metastases (LNM/DM) first. (2) Methods: A total of 303 patients from six institutions (March 1982–February 2015) were combined with individual patient data from a PubMed search, totaling 949 patients. The primary outcome was recurrence patterns. (3) Results: (a) More patients presented with lymph node metastases (LNMs) than DMs at diagnosis: 17.9% (166 among the 929 patients with known staging) vs. 1.9% (18/929); (b) 310/929 (33.4%) developed lifetime DM, of whom 220/310 also developed LNM. The majority (133 patients) of patients were documented to have developed LNM before DM. (c) A shorter median time of 1.5 months (range: 0–47.0) from initial diagnosis to LNM, versus 8 months (range: 0–107.8) to DM, was also found. Another observation was that 2.4% (23/949) of patients with primary tumors ≤1 cm developed lifetime DM, with the smallest being 0.2 cm. (4) Conclusions: Three observations support the idea that prior LNM gives rise to subsequent DM as the main pathway of dissemination in MCC. This implies that patients with nodal metastases should be considered for adjuvant systemic therapy studies as an enriched population. Participation in clinical trials is strongly encouraged.

## 1. Introduction

Merkel cell carcinoma (MCC), a rare skin cancer, has a natural history of developing early distant metastases (DMs) [1,2,3]. It was first described by Toker et al. in 1972 [4]. Primary MCCs appear most frequently in sites with sun exposure: 44% on the head and neck, 28% on the leg, 16% on the arm, and 9% on the buttock. These site distributions have remained similar among older and newer series [5].

The etiological factors for MCC include ultraviolet sun exposure, Merkel cell polyomavirus (MCPyV) infection, and immunosuppression (iatrogenic or other causes) [1]. The cell of origin is related to the above factors. Based upon early histologic/ultrastructural studies, MCC was thought to originate from Merkel cells in the basal layer of the epidermis and hair follicles. These Merkel cells are associated with sensory neurites in the dermal papillae (skin mechanoreceptors) [1,6]. Alternatively, it may originate from an immature multipotent stem cell that acquires neuroendocrine features during malignant transformation to MCC. This explains the similar appearance and immunostaining patterns to other neuroendocrine carcinomas [2]. Around 80% of MCCs are MCPyV-positive (MCPyV+), but the percentage varies with the sensitivity of the detection methods used [7]. These MCCs might arise from mesodermal fibroblasts of the skin dermis and MCPyV-negative (MCPyV-) tumors from ectodermal epidermal keratinocytes, so MCC would be the first cancer that developed from two distinct germ layers: ectoderm and mesoderm [8]. They have different mutation burdens. MCPyV+ tumors are likely true neuroendocrine carcinomas, with a dermal origin, probably from fibroblasts which have been transformed by the integration of the viral genome. MCPyV- tumors are likely derived from either keratinocytes or epidermal stem cells, are probably squamous cell carcinomas with neuroendocrine differentiation, and are related to ultraviolet sun damage. Important prognostic factors include TNM tumor stage, immunosuppression, age, sex, lympho-vascular invasion, and MCPyV status, with MCPyV- tumors or P63 expression having the worse outcomes [1].

MCC has a rising incidence globally and has recently triggered increasing interest among dermatologists and oncologists [1]. The incidence of MCC varies from country to country. Australia has a high rate and a far greater proportion of MCPyV- tumors than places such as the United Kingdom with less sun exposure.

**Figure 1:**
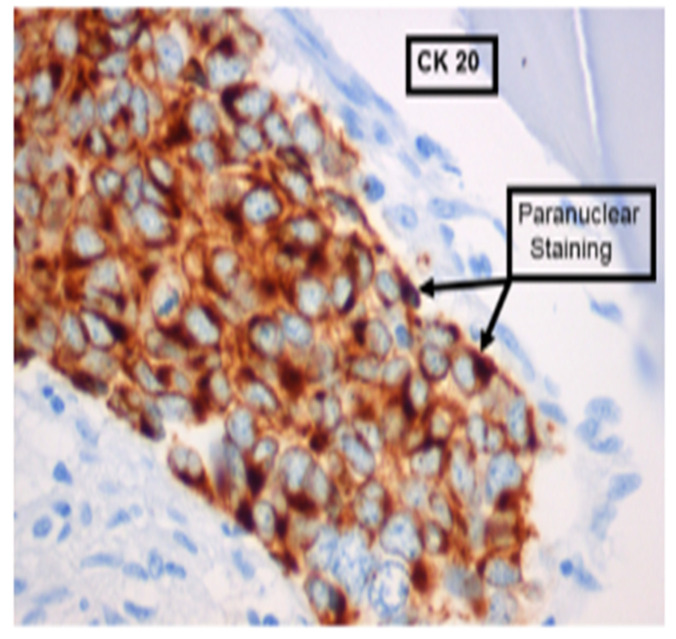
Typical immunochemical staining of Merkel cell carcinoma, curtesy of Professor Michael Veness, showing positive staining for cytokeratin (CK)-20.

Morphologic appearances include small cell size, nuclear molding, scanty cytoplasm (or high nuclear–cytoplasmic ratio), and hyperchromatic nuclei with ’salt and pepper’ chromatin [1]. Its hallmark histology is shown in Figure 1, outlining cytokeratin (CK)-20 positive and negative for both CK7 and thyroid transcription factor-1 [1].

A few large studies have documented conflicting results as to whether LNM is the initial and the most frequent site of recurrence. The pattern of spread was reported in 1991 by Yiengpruksawan et al. from the New York Memorial Sloan-Kettering Cancer Center [9]. A total of 40/70 (87%) patients had lifetime lymph node metastases (LNMs). They concluded that DM was nearly uniformly preceded by LNM and was uniformly fatal despite treatment. In 1996, the Loma Linda University Medical Center reported that LNM was the first site of recurrence in 60% of cases, hence preceding the appearance of DM [10]. The authors suggested that SLNB and radiotherapy and/or node dissection may improve outcomes by decreasing DM. In 1997, Queirolo et al. from the University of Genoa in Italy reported local recurrence in 30% of MCC patients, LNM in 50–65%, and DM in 28–40% [11]. In 2002, Morrison et al. reported that the rate of DM developing from a head-and-neck-located MCC was 36% in the M. D. Anderson Cancer Center, which has the largest experience of MCC in the world [12]. In 2016, Veness et al. reported on 36 published studies (1997–2015) comprising 692 clinically node-negative patients who all underwent sentinel lymph node biopsy (SLNB) [13]. By adding 29 patients treated at Westmead Hospital in Sydney, the large cohort of 721 cases had a 29.6% positive SLNB rate. The authors noted that more DMs were identified after a positive SLNB than negative SLNB (17.6% vs. 7.3%, *p* < 0.001).

Unlike the above studies, a 2013 report from the Massachusetts General Hospital on their 30-year experience showed that the first site of recurrence was distant in 52% of patients, nodal in 27%, and local in 21% [14]. DM occurred in 9% of all patients, with 0% 5-year cause-specific survival. Recurrence occurred in 56% of the 161 patients after a positive SLNB and in 39% of patients with a negative SLNB. Half of these patients recurred at a median time of 9 months [14].

One of the drawbacks from the data of big institutions is the availability of expertise in management and a higher rate of adherence to contemporary treatment guidelines, which are not the case in smaller institutions or developing countries. Their surgeons subspecialize in surgical oncology and are also skillful due to large patient numbers. In addition, the management of MCC, a neuroendocrine cutaneous carcinoma, has changed over time, with the use of (a) PET imaging, (b) better surgical techniques (SLNB, Moh’s micrographic surgery), (c) higher doses of radiotherapy, more advanced radiotherapy treatment techniques to focus on the primary tumor (intensity-modulated radiotherapy, brachytherapy, inverse treatment planning), and (d) the more prevalent use of systemic therapy for definitive or adjuvant treatment, alone or in combination with other treatment modalities [3]. Systemic therapy is usually in the form of chemotherapy or immunotherapy, particularly in the past few years; our research team would like to revisit the issue of the pattern of spread for MCC. Due to controversies in the older literature from large institutions, we sought to document the pattern of recurrence as the primary objective by building our own large database to examine this pattern in both large and small community centers internationally. Data were collected by seven co-authors from three countries: Canada, Australia, and France. The aggregated database enabled the collection of a large dataset, as MCC is a rare tumor, to gather enough patients for analysis. Our eventual goal was to identify the high-risk group(s) who may potentially benefit the most from neoadjuvant or adjuvant systemic therapy, while sparing patients at lower risk. In 2023, a trial was published using adjuvant immunotherapy with nivolumab versus observation in the completely resected MCC (ADMEC-O) trial, which demonstrated that adjuvant nivolumab increased disease-free survival (DFS), although the overall survival (OS) result is still immature [15].

## 2. Materials and Methods

Patient data from six jurisdictions from France, Canada, and Australia were collected retrospectively from both paper and electronic charts. More individual patient data were added after a PubMed literature search. A Microsoft excel datasheet was designed. There were no exclusion criteria for patients. The following were compiled for patients after ethics approval: baseline information of age, sex, initial clinical and pathological stages, site, time delay before seeing doctors, other concurrent tumor(s), maximum dimension of the primary tumor, LNM and DM, histological details, and history of immunosuppression/co-morbidities/previous radiotherapy. We recorded the treatment(s) received: surgery (e.g., Mohs microsurgery, nodal dissection or excision alone, and resection margin), radiotherapy (doses, field coverage, response), chemotherapy (specific chemotherapy drugs, number of cycles, response), and outcomes such as recurrence (timing, site, and subsequent treatments) and final disease status.

To our knowledge, our MCC database is one of the most comprehensive in the literature; details of it will be covered in the Discussion Section. MCC polyomavirus status was not included due to non-availability at the time of study. Follow-up timing varied among the different centers and was based on the judgment of each clinician according to the assessed recurrence risk since no recommended internationally accepted follow-up guidelines were available at the time of this study.

### Statistical Analysis

Pattern of recurrence was analyzed as the primary outcome, with survival rate as a secondary outcome. CSS was defined as the time interval from diagnosis to death from MCC or censored at the last follow-up date if the patient was still alive at the time of analysis. OS was defined as the time interval from diagnosis to death regardless of the cause, or last follow-up date for censoring, as described above. The Kaplan–Meier method was used to generate survival curves [16]. The Cox Proportional Hazards model was used to identify risk factors for DM [17].

## 3. Results

In total, 303 patients presented to our respective cancer centers in Canada, France, and Australia from March 1982 to Feb 2015. No patients were excluded. They were staged accordingly by computerized axial tomography (CT) imaging, with or without bone scans. Positron emission tomography (PET) imaging was ordered for selected patients. The median FU was 21.8 (range, 0.5–264.8) months. The head and neck were the primary site of disease in 44.3% (35/79) of patients; the limbs in 36.7% (29/79); and the body in 21.5% (17/79, Table 1). Ten percent (8/79) of patients presented as LNM with an unknown primary. The median dimension of the primary was 2.5 cm (range, 0.2–17.0 cm); and 8.9% (7/79) were ≤1 cm in size, with the smallest being 0.2 cm.

The data on patterns of spread are noted below: (a) A total of 59/303 (19.5%) patients presented with LNM, but only 3/303 (1.0%) with DM. The above patient number included one patient who presented with both LNM and DM at the same time. In five patients, the sequence of developing LNM and DM was unclear. (b) Altogether, 79/303 (26.1%) patients developed DM within their lifetime, the focus of this study (Table 1). Among these 79 patients with DM, 47/79 (59.4%) also developed LNM at different times: 31/47 (66.0%) before DM, 10/47 (21.3%) within a month of DM, and 1/47 (2.2%) after DM diagnosis. (c) The median time between the self-discovery of skin spot(s) and the first doctor’s visit was 3.0 months (range, 0.5–36.0 months). The median time from cancer diagnosis to LNM was 1.5 months (range, 0–47.0 months), and from cancer diagnosis to DM, it was 8 months (range, 0–107.8 months).

**Figure 2:**
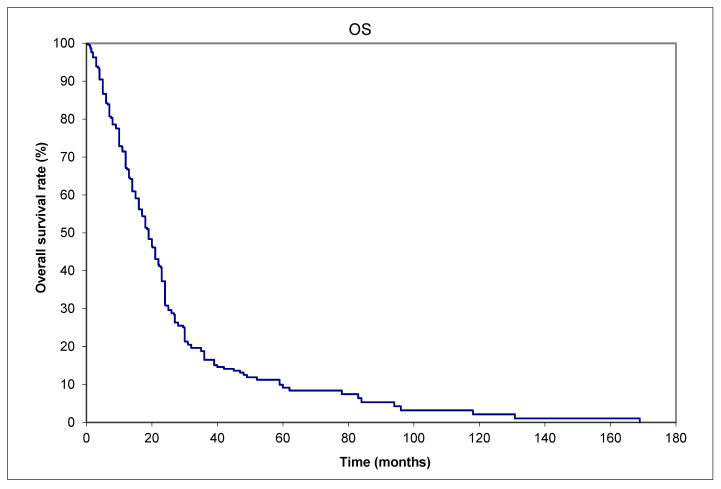
Merkel cell carcinoma. OS (overall survival) of 79 patients with distant metastases from the time of initial diagnosis to death from any cause.

**Figure 3:**
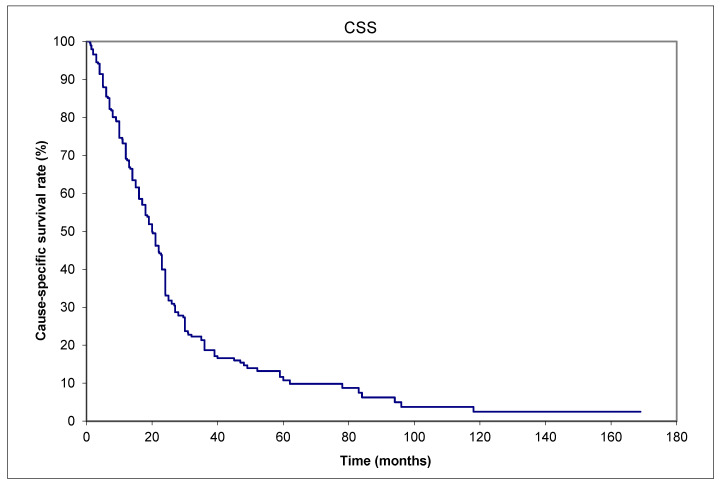
Merkel cell carcinoma. CSS (cause-specific survival) of 79 patients with distant metastases from the time of initial diagnosis to death from Merkel cell carcinoma.

Despite different treatments, as listed in Table 2, most patients eventually died of the cancer. The 1-, 2-, and 5-year OS rates were 56.5%, 30.4%, and 9.2%, respectively (Figure 2). The 1-, 2-, and 5-year CSS rates were 56.5%, 32.1%, and 11.6%, respectively (Figure 3). Table 3 shows the risk factors for DM.

The analysis has been verified by our team on the entire database of 949 patients, i.e., 646 individual patient data were added after a PubMed search (with 730 stage I + II, 176 stage III, 22 stage IV, and 21 unknowns at diagnosis). A total of 310/949 developed lifetime DM. Their treatments were surgery in 80.6% (250/310) of patients with or without adjuvant treatments. Among these 250 patients, combined modality treatments were given to 77/250 patients, of which 24 received adjuvant chemotherapy. Radiotherapy alone was given to 37/310 patients, chemotherapy alone to 9, it was unknown in 9, and none in 5.

A total of 23/949 (2.4%) patients with maximum primary dimension ≤ 1 cm developed distant metastases (DMs) in their lifetime, including a 0.2 cm primary. The median size of the primary tumor was 2.5 cm (range: 0.2–17.0 cm), for LNM the median size was 4 (range: 0.3–13.0) cm, and for DM the median size was 2.6 cm (range: 1.0–15.0 cm). Similar trends of observations to deduce the pattern of spread are as follows: (a) more patients were diagnosed with nodal rather than DM among the 929 patients with known clinical stage: 17.9% (166/929) vs. 1.9% (18/929); (b) 310/929 (33.4%) patients developed lifetime DM, of which 220 also developed lifetime LNM. Among those with known disease status time, the majority (133 patients) were documented to develop LNM before DM.

## 4. Discussion

Our results are comparable to other centers in terms of site location and the rate of lifetime development of DM: 79/303 (25%), with the 5-year OS and CSS rates both approximately 10%, respectively. OS and CSS were similar, implying that most patients died from their MCC [18,19,20,21]. Regarding low five-year survival rates, which may be a result of limited access to modern therapies, the patients in the survival plots were a subset of all patients. They developed lifetime DM, possibly biologically more aggressive compared to others at the same stages. They do not represent the whole 949-patient database (with 730 stage I + II, 176 stage III, 22 stage IV, and 21 unknown stage at diagnosis). Modern SLNB or PET scans can apparently improve outcomes since clinically node-negative cases identified by conventional imaging in the past are now reclassified as node-positive (stage migration or the Will Rogers phenomenon) [22]. With under-treatment, our data may reflect the *natural history* of MCC better to demonstrate the pattern of spread.

The findings of the Massachusetts General Hospital are contrary to the results of the present study with international data and those from American institutions such as New York Memorial Sloan-Kettering, Loma Linda, and the University of Genoa in Italy [9,10,11,14]. Massachusetts General Hospital is a major tertiary center [8]. The initial stage distribution of the 161 patients was 56% localized, 35% nodal, and 9% distant. The initial recurrences were in distant sites in 52% of cases, compared to 27% in nodal sites and 21% in local sites. The comparatively higher proportion of patients presenting with stage III (nodal disease) may imply that they were further along in their disease course, leading to more distant spread, as seen in the majority of their recurrences.

Our study focused on 303 patients from six jurisdictions in Canada, France, and Australia. The results were verified by checking the entire 949-patient database, which included 643 individual patient data points added from the literature. Our stage distribution was 77% localized disease, 19% nodal, 2% distant, and 2% unknown. These differences may account for the varying patterns of spread documented by researchers.

With regard to the controversies surrounding the pattern of spread, the three observations support the hypothesis that LNM leads to the subsequent development of DM as the main pathway of dissemination. We found that the majority of patients with LNM were documented before the development of DM. This, together with the higher proportion of patients presenting with LNM than DM, and the shorter time interval from initial diagnosis to LNM all point toward nodal disease preceding DM, or the so-called “cascade theory” of dissemination. The word “cascade” means something arranged or occurring in a series or in a succession of stages [23]. According to the cascade theory, the initial spread is to nodes, followed by distant metastases, representing one route by which cancer cells can metastasize. By studying the metastatic cascade, a combination of systemic therapies can be administered to prevent distant metastases [24].

We identified 23/949 (2.4%) patients with a small ≤1 cm primary tumor, and in particular, a 0.2 cm primary later developed DM. This finding illustrates that even small primary tumors have the potential to metastasize, in conflict with some older guidelines tending to favor postoperative observation in these small primaries. The latest MCC guidelines of the National Comprehensive Cancer Network in 2024 no longer have this recommendation [21].

Patients with locoregional disease may benefit from neoadjuvant or adjuvant treatment(s). Recently, immunotherapy has emerged as a likely beneficial adjuvant treatment as well as a palliative treatment modality [25]. Immunotherapy confers about a 50% durable benefit in metastatic MCC as a first-line therapy and 30% as a second-line therapy. Adverse events with immunotherapy are not as frequent as with patients receiving cytotoxic chemotherapy (e.g., carboplatin and etoposide) [26]. Chemotherapy provides only short-lived responses (2–3 months) and is notably toxic in many patients, resulting in 3.4% dying a treatment-related death [27].

The limitations of this study are as follows: (a) The use of multi-center retrospective data from the year 1982 to 2015, and the fact that the treatment paradigm has changed over the years. Survival/recurrence outcomes may be different due to the more current use of PET imaging, sentinel node biopsy, immunotherapy, and neoadjuvant treatments in addition to radiotherapy. The data are the closest we can obtain regarding the natural history of MCC in the chemotherapy era before the introduction of immunotherapy. Our report is an observational retrospective study reporting real-world data from many international countries, including those with poor healthcare resources, which may not be able to afford many (or any) SLNB procedures. If SLNBs were performed in all cases, the actual number of patients presenting with nodal metastases would be even higher, thus strengthening our conclusion that nodal metastases are more common than distant metastases at diagnosis, indicating that nodal metastases likely represent earlier events in the course of MCC.

(b) Data are heterogeneous and center-dependent. Prospective data are difficult to obtain for this rare cancer. In fact, different practices of various centers enhance the generalizability of the findings, but they potentially introduce bias. The follow-up timing varied according to each center due to a lack of international follow-up guidelines at the time of this study. Data were collected by seven co-authors from three countries: Canada, Australia, and France. These represent two of the three western Prairie provinces (Alberta and Saskatchewan), a small portion of Ontario (Windsor, London, and close-by regions), and relatively small areas of France and Australia. These cannot reflect the practice of their respective countries as other regions may have more healthcare funding.

(c) Another limitation is the lack of data on MCC polyomavirus status, which could impact prognosis and treatment response. This affects the early detection of recurrence by the MCPyV antibody titer used in modern times.

(d) There are missing data, especially for cases in the literature on high-risk factors associated with local and distant recurrence such as lympho-vascular invasion, margin status, and association with immunosuppression. Missing data may introduce bias, but these only constitute 2% of the entire database and are unlikely to affect the conclusion of this study regarding the pattern of spread for MCC.

The strengths of this study are our detailed database being more comprehensive than many others in the literature, due to the multidisciplinary collaboration. Our database contains more details than larger published reports, e.g., Pan-Canadian Merkel Cell Cancer Collaborative [28], the American National Cancer Database (NCDB) [29], and the Surveillance, Epidemiology, and End Results (SEER) program [30]. All stages of MCC patients were included without any bias, whether they were treatment-naïve or previously treated elsewhere, although we stratified them at the time of analysis.

Lastly, to summarize and elaborate on the current standard of care from the literature, our research team advocates the following:

(a) After initial diagnosis, patients with primaries of any size should be referred without delay to experienced specialist(s) for definitive treatment, in a tertiary center if possible, since delay in surgery or postoperative radiotherapy was documented to be associated with worse outcome [31,32]. The Fred Hutchinson Cancer Center in Seattle reported on 124 MCC patients with a median time to initiate postoperative radiotherapy (ttPORT) of 41 days (range: 8–125 days) [31]. The median follow-up was 55 months. In total, 17 (14%) patients experienced a locoregional recurrence (LRR), 14 (82%) of which arose outside the radiation field. LRR at 5 years was increased for ttPORT > 8 weeks vs. ≤ 8 weeks, 28.0% vs. 9.2%, *p* = 0.006. There was an increase in the cumulative incidence of cancer-specific death with increasing ttPORT (Hazard Ratio = 1.14 per 1-week increase, *p* = 0.016) [31]. Readers should note that different cutoffs for surgical delays were used in the two above references [28,29,31,32], so currently there is no definite recommended guideline for the optimal time to start treatment. Surgeons are advised to perform wide local excision or Moh’s microsurgery to achieve negative margins but avoid extensive reconstructions that require a longer time to heal and hence delay radiotherapy initiation, as per the NCCN recommendations [21].
(b) Multidisciplinary discussions of the best treatment options should be made on tumor boards. As combination therapy could have more side effects, a consideration of co-morbidities, performance status, and the available social support during and after the treatment for the patient is needed. Fine judgment is necessary to decide if the benefits outweigh the risks in elderly patients.
(c) Clinicians should constantly update their knowledge by looking at websites such as merkelcell.org [33], Fred Hutchison Research Cancer Center [34], Memorial Sloan-Kettering Cancer Center [35], Cleveland Clinic [36], Mayo Clinic [37], the Skin Cancer Foundation [38], and NCCN (National Comprehensive Cancer Network guidelines [21]. More recently, retifanlimab has emerged as a notably effective treatment strategy, leading to US Food and Drug Administration Accelerated Approval for metastatic or recurrent locally advanced MCC based on the phase II POD1UM-201 study [39,40].
(d) Enrollment in clinical trials is strongly encouraged. The results of the recent phase II study ADMEC-O could be confirmed with more trials to encourage clinicians to consider adopting adjuvant nivolumab into their clinical practice. Hypofractionated radiotherapy research is ongoing as well [41].
(e) More research on cancer prevention and the development of treatment resistance should be performed in the future. An example is the study of tumor-associated macrophages (TAMs) in MCC, and the association of S100A8-expressing TAMs with resistance to anti-PD-L1 inhibitors (where PD-L1 stands for programmed death-ligand 1). Silk et al. commented that these data improve our understanding of why some tumors with brisk tumor-infiltrating lymphocytes do not respond to immunotherapy [42]. Another study on MCPyV+ metastatic MCCs treated with an intra-tumoral stimulator of interferon genes (STING) agonist (ADU-S100) plus intravenous anti-PD-1 antibody (spartalizumab) attained a durable objective response with the regression of both injected and non-injected lesions [43]. Eventually, a cancer vaccine may be possible [44].
(f) Further analysis of existing data in well-established cancer clinics and the literature can help to resolve many puzzles for MCC on the patterns and risk factors of recurrence, patterns of spread, radiation dose–response relationship, etc. [45,46,47].

These ideas are relevant to all MCC patients and clinicians. The basic observations made in the present study are especially important in developing countries with inadequate staging resources for patient management and may become a useful tool for other cancers as well.

## 5. Conclusions

Our study showed that the development of LNM, leading to the subsequent development of DM, is the main pathway of dissemination. This implies that patients with nodal metastases should be considered for adjuvant systemic therapy studies as an enriched population. Participation in clinical trials is strongly encouraged.

## Figures and Tables

**Table 1:** Merkel cell carcinoma patient characteristics: a total of 79 patients from six centers developed distant metastases in their lifetime.

Aggregated data from	Saskatchewan (Canada)			13 (16%)	
	Alberta (Canada)			17 (22%)	
	London, Ontario (Canada)			15 (19%)	
	Windsor/Ontario, Canada			5 (6%)	
	Amiens (France)			9 (11%)	
	Westmead, New South Wales (Australia)			20 (25%)	
Baseline characteristics	Age:			median 78 (range: 47–95) years	
	Sex:			29 males and 50 females	
	Size of primary tumor:			median 2.5 (range: 0.2–17) cm	
Initial stages		Local	Nodal	Distant metastases	Unknown
	Clinical	42 (53%)	28 (35%)	8 (10%)	1 (1%)
	Pathological	35 (44%)	35 (44%)	8 (10%)	1 (1%)
Primary site	Head and neck			35 (44%)	
	Limb (upper or lower)			23 (29%)	
	Trunk			13 (16%)	
	Unknown primary, presented with nodes only			8 (10%)	
Timing of nodal metastases (patient number = 47)	Before distant metastases diagnosis			31/47 (66%)	
	Within 1 month of distant metastases diagnosis			10/47 (21%)	
	After distant metastases diagnosis			1/47 (2%)	
	Unknown time relative to distant metastases			5/47 (11%)	

**Table 2:** Merkel cell carcinoma with distant metastases: treatment and outcome of the 79 patients from six centers.

Treatment of localized disease at presentation (patient number = 43)	Surgery	24/43 (56%)
	Surgery + Radiotherapy	11/43 (26%)
	Surgery + Chemotherapy	1/43 (2%)
	Radiotherapy alone	4/43 (9%)
	Radiotherapy + Chemotherapy	1/43 (2%)
	None	2/43 (5%)
Treatment of nodal metastases at presentation (patient number = 28)	Surgery	6/28 (21%)
	Surgery + Radiotherapy	7/28 (25%)
	Surgery + Radiotherapy + Chemotherapy	2/28 (7%)
	Radiotherapy alone	13/28 (46%)
Treatment of distant metastases at presentation (patient number = 8)	Radiotherapy + Chemotherapy	3/8 (38%)
	Chemotherapy alone	2/8 (25%)
	None	3/8 (38%)
Final vital status	Alive	8/79 (10%)
	Dead	71/79 (90%)
Cause of death among those expired (patient number = 71)	Merkel cell carcinoma	65/71 (92%)
	Intercurrent disease	6/71 (8%)

**Table 3:** Merkel cell carcinoma: risk factors for distant metastases with adjusted Cox Proportional Hazards model.

	Variable	Hazard Ratio	(95% Confidence Interval)	*p* Values
**Age:**	60	Reference variable		
	70	0.90	(0.64–1.26)	0.50
	80	1.06	(0.66–1.68)	0.82
	90	1.75	(0.89–3.46)	0.11
**Sex:**	Male	0.87	(0.54–1.42)	0.59
	Female	Reference variable		
**Chemotherapy:**	Yes	0.56	(0.19–1.62)	0.29
	No	Reference variable		
**Clinical stage:**	Localized disease	2.53	(1.21–5.28)	**0.013**
	Primary ≤1 cm	Reference variable		
	Primary >1 cm	1.32	(0.61–2.89)	0.49
	Nodal metastases	3.27	(1.85–5.78)	**<0.001**
	Distant metastases	21.42	(7.15–64.21)	**<0.001**
**Previous irradiation:**	Yes	2.95	(0.90–9.61)	0.073
	No	Reference variable		

## Data Availability

Data are unavailable to the public due to privacy (restrictions by the Ethics Committee above).

## Abbreviations

The following abbreviations are used in this manuscript:

ADMEC-O	Adjuvant immunotherapy with nivolumab versus observation
ADT	Androgen deprivation therapy
CK	Cytokeratin
CSS	Cause-specific survival
CT	Computerized tomography
DFS	Disease-free survival
DMs	Distant metastases
LNMs	Lymph node metastases
LRR	Locoregional recurrence
MCC	Merkel cell carcinoma
NCDB	National Cancer Database
OS	National Comprehensive Cancer Network
NCCN	Overall survival
PD-L1	Programmed death-ligand 1
PET	Positron emission tomography
PFS	Progression-free survival
SEER	Surveillance, Epidemiology, and End Results (SEER) program
SLNB	Sentinel lymph node biopsy
STING	Stimulator of interferon genes
TAMs	Tumor-associated macrophages
ttPORT	Time to initiate postoperative radiotherapy

## References

[1] Tai P., Park S.Y., Nghiem P.T., Canellos G.P., Schnipper L. (2025). Pathogenesis, clinical features, and diagnosis of Merkel cell (neuroendocrine) carcinoma. UpToDate.

[2] Tilling T., Moll I. (2012). Which are the cells of origin in Merkel cell carcinoma?. J. Skin Cancer.

[3] Tai P., Au J. (2018). Skin cancer management—Updates on Merkel cell carcinoma. Ann. Transl. Med..

[4] Tang C.K., Toker C., Nedwich A., Zaman A.N. (1982). Unusual cutaneous carcinoma with features of small cell (oat cell-like) and squamous cell carcinomas. A variant of malignant Merkel cell neoplasm. Am. J. Dermatopathol..

[5] Raaf J.H., Urmacher C., Knapper W.K., Shiu M.H., Cheng E.W. (1986). Trabecular (Merkel cell) carcinoma of the skin. Treatment of primary, recurrent, and metastatic disease. Cancer.

[6] Ratner D., Nelson B.R., Brown M.D., Johnson T.M. (1993). Merkel cell carcinoma. J. Am. Acad. Dermatol..

[7] Sunshine J.C., Jahchan N.S., Sage J., Choi J. (2018). Are there multiple cells of origin of Merkel cell carcinoma?. Oncogene.

[8] Nirenberg A., Steinman H., Dixon J., Dixon A. (2020). Merkel cell carcinoma update: The case for two tumours. J. Eur. Acad. Dermatol. Venereol..

[9] Yiengpruksawan A., Coit D.G., Thaler H.T., Urmacher C., Knapper W.K. (1991). Merkel cell carcinoma. Prognosis and management. Arch. Surg..

[10] Victor N.S., Morton B., Smith J.W. (1996). Merkel cell cancer: Is prophylactic lymph node dissection indicated?. Am. Surg..

[11] Queirolo P., Gipponi M., Peressini A., Raposio E., Vecchio S., Guenzi M., Sertoli M.R., Santi P., Cafiero F. (1997). Merkel cell carcinoma of the skin. Treatment of primary, recurrent and metastatic disease: Review of clinical cases. Anticancer Res..

[12] Gillenwater A.M., Hessel A.C., Morrison W.H., Burgess M., Silva E.G., Roberts D., Goepfert H. (2001). Merkel cell carcinoma of the head and neck: Effect of surgical excision and radiation on recurrence and survival. Arch. Otolaryngol. Head Neck Surg..

[13] Gunaratne D.A., Howle J.R., Veness M.J. (2016). Sentinel lymph node biopsy in Merkel cell carcinoma: A 15-year institutional experience and statistical analysis of 721 reported cases. Br. J. Dermatol..

[14] Santamaria-Barria J.A., Boland G.M., Yeap B.Y., Nardi V., Dias-Santagata D., Cusack J.C. (2013). Merkel cell carcinoma: 30-year experience from a single institution. Ann. Surg. Oncol..

[15] Becker J.C., Ugurel S., Leiter U., Meier F., Gutzmer R., Haferkamp S., Zimmer L., Livingstone E., Eigentler T.K., Hauschild A. (2023). Adjuvant immunotherapy with nivolumab versus observation in completely resected Merkel cell carcinoma (ADMEC-O): Disease-free survival results from a randomised, open-label, phase 2 trial. Lancet.

[16] Kaplan E.L., Meier P. (1958). Non-parametric estimation from incomplete observations. J. Am. Stat. Assoc..

[17] Cox D.R. (1972). Regression Models and Life-Tables. J. R. Stat. Soc. Ser. B.

[18] Coggshall K., Tello T.L., North J.P., Yu S.S. (2018). Merkel cell carcinoma: An update and review: Pathogenesis, diagnosis, and staging. J. Am. Acad. Dermatol..

[19] Tai P., Park S.Y., Nghiem P.T., Silk A., Canellos G.P., Schnipper L. (2024). Staging and treatment, and surveillance of locoregional Merkel cell carcinoma. UpToDate.

[20] Park S.Y., Nghiem P.T., Tai P., Silk A., Canellos G.P., Schnipper L. (2024). Treatment of recurrent and metastatic Merkel cell carcinoma. UpToDate.

[21] National Comprehensive Cancer Network (NCCN) Guideline Website. www.nccn.org.

[22] Ginsburg K.B., Bell S., Bukavina L., Schober J.P., Magee D., Kutikov A. (2022). The Phenomenon of “Therapeutic” Nodal Yield at Cystectomy for Bladder Cancer: Do Not Discount the Will Rogers Effect. Eur. Urol. Open Sci..

[23] CASCADE English Meaning—Cambridge Dictionary. https://dictionary.cambridge.org/us/dictionary/english/cascade.

[24] Miranda I., Jahan N., Shevde L.A. (2025). The metastatic cascade through the lens of therapeutic inhibition. Cell Rep. Med..

[25] Kaufman H.L., Russell J.S., Hamid O., Bhatia S., Terheyden P., D’Angelo S.P., Shih K.C., Lebbé C., Milella M., Brownell I. (2018). Updated efficacy of avelumab in patients with previously treated metastatic Merkel cell carcinoma after ≥1 year of follow-up: JAVELIN Merkel 200, a phase 2 clinical trial. J. Immnother. Cancer.

[26] Kaufman H.L., Russell J., Hamid O., Bhatia S., Terheyden P., D’Angelo S.P., Shih K.C., Lebbé C., Linette G.P., Milella M. (2016). Avelumab in patients with chemotherapy-refractory metastatic Merkel cell carcinoma: A multi-center, single-group, open-label, phase 2 trial. Lancet Oncol..

[27] Tai P.T.H., Yu E., Winquist E., Hammond A., Stitt L., Tonita J., Gilchrist J. (2000). Chemotherapy in neuroendocrine Merkel cell carcinoma of the skin: Case series and review of 204 cases. J. Clin. Oncol..

[28] Nayak A.L., Pickett A.T., Delisle M., Dingley B., Mallick R., Hamilton T., Stuart H., Talbot M., McKinnon G., Jost E. (2023). Survival of Patients With Head and Neck Merkel Cell Cancer: Findings From the Pan-Canadian Merkel Cell Cancer Collaborative. JAMA Netw. Open.

[29] Yusuf M.B., Gaskins J., Wall W., Tennant P., Bumpous J., Dunlap N. (2020). Immune status and the efficacy of radiotherapy on overall survival for patients with localized Merkel cell carcinoma: An analysis of the National Cancer Database. J. Med. Imaging Radiat. Oncol..

[30] Nudelman N.T., Ekhator N., Rothschild M., Wladis E.J. (2024). A SEER program study of survival trends in Merkel cell carcinoma of the eyelid: 2000-2019. Orbit.

[31] Alexander N.A., Schaub S.K., Goff P.H., Hippe D.S., Park S.Y., Lachance K., Bierma M., Liao J.J., Apisarnthanarax S., Bhatia S. (2024). Increased risk of recurrence and disease-specific death following delayed postoperative radiation for Merkel cell carcinoma. J. Am. Acad. Dermatol..

[32] Desai A.D., Behbahani S., Samie F.H. (2022). Predictors of time to definitive surgery and survival in Merkel cell carcinoma: Analysis of the US National Cancer Database. Clin. Exp. Dermatol..

[33] Clinical trials for Merkel Cell Carcinoma. https://merkelcell.org/treatment/clinical-trials/.

[34] https://www.fredhutch.org.

[35] https://www.mskcc.org.

[36] https://my.clevelandclinic.org.

[37] https://www.mayoclinic.org.

[38] https://www.skincancer.org.

[39] touchONCOLOGY-D-23-00021.pdf. https://touchoncology.com/wp-content/uploads/sites/2/2024/03/touchONCOLOGY-D-23-00021.pdf.

[40] Grignani G., Rutkowski P., Lebbe C., Guida M., Marqueste C.G., De Braud F.G.M., Spagnolo F., Burgess M., Montaudie H., Depenni R. (2023). 1146P Updated results from POD1UM-201: A phase II study of retifanlimab in patients with advanced or metastatic Merkel cell carcinoma (MCC). Ann. Oncol..

[41] Treatment Clinical Trials for Merkel Cell Cancer—NCI. https://www.cancer.gov/research/participate/clinical-trials/disease/merkel-cell/treatment.

[42] Silk A.W., Davar D. (2024). Tumor-Associated Macrophages in Merkel Cell Carcinoma: Old Balances, New Checks. Clin. Cancer Res..

[43] Pulliam T., Jani S., Goff P.H., Bhakuni R., Tabachnick-Cherny S., Smythe K., Seaton B.W., Tachiki L., Kulikauskas R., Church C. (2024). Intratumoral STING agonist reverses immune evasion in PD-(L)1-refractory Merkel cell carcinoma: Mechanistic insights from detailed biomarker analyses. J. Immunother. Cancer.

[44] Tabachnick-Cherny S., Pulliam T., Church C., Koelle D.M., Nghiem P. (2020). Polyomavirus-driven Merkel cell carcinoma: Prospects for therapeutic vaccine development. Mol. Carcinog..

[45] Joseph K., Wong J., Abraham A., Zebak J., Patel A., Jones Thachuthara A., Iqbal U., Pham T.M., Menon A., Ghosh S. (2022). Patterns and predictors of relapse in Merkel cell carcinoma: Results from a population-based study. Radiother. Oncol..

[46] Park S.Y., Tai P., Assouline A., Koul R., Dubey A., Lian J., Yu E., Veness M., Joseph K. (2024). Merkel cell carcinoma (MCC) of the skin: Comprehensive analysis of radiation (RT) doses of aggregate patient data from literature. Radiother. Oncol..

[47] Joseph K., Tai P., Veness M., Lian J., Assouline A., Koul R., Dubey A., Park S.Y., Yu E. (2024). Merkel cell carcinoma (MCC) of the skin: When is local radiotherapy (RT) without nodal coverage adequate? Comprehensive analysis of aggregate patient data from literature. Radiother. Oncol..

